# Predicting Mammogram Screening Follow Through with Electronic Health Record and Geographically Linked Data

**DOI:** 10.1158/2767-9764.CRC-23-0263

**Published:** 2023-10-19

**Authors:** Matthew Davis, Kit Simpson, Leslie A. Lenert, Vanessa Diaz, Alexander V. Alekseyenko

**Affiliations:** 1 Department of Public Health Sciences, College of Medicine, Medical University of South Carolina, Charleston, South Carolina.; 2 Department of Healthcare Leadership and Management, College of Health Professions, Medical University of South Carolina, Charleston, South Carolina.; 3 Biomedical Informatics Center, Medical University of South Carolina, Charleston, South Carolina.; 4 Department of Internal Medicine, College of Medicine, Medical University of South Carolina, Charleston, South Carolina.; 5 Department of Family Medicine, College of Medicine, Medical University of South Carolina, Charleston, South Carolina.

## Abstract

Cancer is the second leading cause of death in the United States, and breast cancer is the fourth leading cause of cancer-related death, with 42,275 women dying of breast cancer in the United States in 2020. Screening is a key strategy for reducing mortality from breast cancer and is recommended by various national guidelines. This study applies machine learning classification methods to the task of predicting which patients will fail to complete a mammogram screening after having one ordered, as well as understanding the underlying features that influence predictions. The results show that a small group of patients can be identified that are very unlikely to complete mammogram screening, enabling care managers to focus resources.

**Significance:**

The motivation behind this study is to create an automated system that can identify a small group of individuals that are at elevated risk for not following through completing a mammogram screening. This will enable interventions to boost screening to be focused on patients least likely to complete screening.

## Introduction

Cancer is the second leading cause of death in the United States, and breast cancer is the fourth leading cause of cancer-related death, with 42,275 women dying of breast cancer in the United States in 2020 (1). This study Predicting Mammogram Screening Follow Through uses machine learning to identify whether patients will complete a mammogram, so that interventions to increase screening adherence can be focused on patients least likely to complete screening.

According to the American Cancer Society, 1 in 8 women nationally will have a diagnoses of breast cancer in their lifetimes (2). Screening is a key strategy for reducing mortality from breast cancer and is recommended by various national guidelines. When localized breast cancer is detected, five-year survival is approximately 99%. However, if it is detected at a regional stage, where cancer has spread to nearby structures or lymph nodes, five-year survival drops to 86% (3). The 2019 National Health Interview Survey (NHIS), CDC/NCHS estimated that cancer screening uptake for females ages 50–74 was 76.2%, well short of the Healthy People 2030 goal of a screening rate of 80.5% (4). In South Carolina, Department of Health and Environmental Control (DHEC) reported during 2013–2017 a breast cancer incidence rate of 129.9 per 100,000 in women, higher than the national average of 125.2 (5). The mortality rate due to breast cancer during the same period for South Carolina was 21.5, higher than the national average of 20.3 per 100,000 women (5).

Mammography screening is recommended every other year by the U.S. Preventive Services Task Force (USPSTF) for women between age of 50 to 74 (6). The USPSTF cites evidence of reduction in a relative risk of mortality from breast cancer as a basis for its recommendation (6). Increasing follow through with recommended screenings can further improve the population level risk. To maximize screening, it is essential to design interventions directed at subpopulations least likely to complete their recommended screenings. To identify this subpopulation, we conducted a retrospective observational study to design a model that predicts timely completion of a mammogram breast cancer screening test after having one ordered by a health care provider, based on demographics, clinical visit data, insurance coverage, and geographic data. Our goal is to design a highly predictive supervised machine learning model, which is both explainable and transparent to aid in intervention design. Our intent is to automate the identification of patients most at risk of not following through using data from an electronic health record (EHR), so that health systems can focus efforts on patients needing follow-up assistance.

The literature addressing mammogram screening uptake has generally focused on two methods, population-level analysis and patient surveys. Heller and colleagues (2018) showed positive correlation between having some college education and county level mammogram uptake for Medicare recipients aged 67–69 (7). The authors reported an inverse correlation between screening uptake and age adjusted mortality, moderated by the ratio of white and black residents in a county (7). Factors affecting mammogram screening uptake were addressed in a 2020 meta-analysis of studies by Özkan and Taylan assessing barriers and behaviors to breast cancer screening across 22 countries (8). The authors report that many factors contribute to lower screening uptake including fear, embarrassment, lack of education of the benefits, socioeconomic challenges as well as health insurance, and accessibility (8). While machine learning has been used extensively to address breast cancer diagnoses, we could not identify any published papers that used machine learning to improve mammography screening uptake. The successful use of machine learning to develop a system to enable automated identification of patients who are unlikely to follow through on screening tests, has the potential for improving screening completion, and thus cancer survival. Interventions designed to increase uptake could then be directed, with the goal of increasing uptake and decreasing the burden of mortality due to breast cancer.

Supervised machine learning provides a set of tools to predict and understand patterns in data and has been applied to a wide variety of health care predictions problems. This approach relies on using known historic outcomes to train a model to make inferences about instances in which the outcome is unknown. A key step to supervised machine learning is data preparation where feature selection takes place. This involves selecting a restricted subset of features from the original large feature set where only useful features are retained, and noninformative features are disregarded. Feature selection methods strive to reduce the number of features, improve accuracy and robustness of the model, and reduce overfitting attributed to noisy variables. Evaluation of a supervised machine learning model involves splitting data into two datasets; a *training set* used for learning the parameters of the model, while a *testing set* is used only for analysis of performance of the trained model. These processes can be repeated across different splits of the original data in a process called cross validation that trains and evaluates a model for a data splitting scenario, resulting in a distribution of performance statistics that estimate how the model will perform on new data.

## Materials and Methods

### Population Selection

Electronic Health Record (EHR) data were sourced from Medical University of South Carolina's patient population as de-identified records. Variables included basic visit information, comorbidity, insurance coverage, and screening test completion information. These data selected female patients, age (50–74) at the time of an outpatient visit, with at least one billed visit during the 2016–2019 timeframe. Controls were defined as having a mammogram test ordered and having had test status of completed within 180 days after the first date it was ordered. Cases were defined as having had a mammogram test ordered, but not completed, billed or with status sent, within 180 days of the first ordered date. The 180-day span was chosen to ensure timeliness of the test completion. Patients with a history or condition code indicating cancer were excluded. Study population demographics are shown in Table 1.

**TABLE 1:** Study population demographics

Variable	Value level	Cases	Controls	*P* ^a^
Race	**White-Caucasian**	576 (59.9%)	482 (55.7%)	0.07
	Race Other	30 (3.1%)	23 (2.7%)	0.58
	**African-American**	355 (36.9%)	360 (41.6%)	**0.04**
Marital Status	**Not Married**	509 (53.0%)	417 (48.2%)	**0.04**
	**Married**	452 (47.0%)	448 (51.8%)	**0.04**
Age	**Age [55–60)**	417 (43.4%)	292 (33.8%)	**<10^−^^4^**
	Age [60–65)	215 (22.4%)	181 (20.9%)	0.46
	Age [65–70)	222 (23.1%)	217 (25.1%)	0.32
	**Age [70–74]**	107 (11.1%)	175 (20.2%)	**<10^−^^7^**

^a^Fisher exact test for variable value level vs. all other levels. *P* < 0.05 values are in bold.

### Feature Construction

Patient-level data were joined by 11-digit FIPS codes to the CDC's 2018 Social Vulnerability Index, creating a rich geographically linked feature-set describing the context where each patient lives (9). Relative tract percentiles that make up the socioeconomic, household composition-disability, minority-status-language and housing-type-transportation themes were used as features. Billed ICD10CM codes prior to the first mammogram ordered were mapped to Elixhauser Comorbidities v2022–1 using python3.8 (10). Features were built using binary encoding of ICD10CM base codes billed before the first order of a mammogram screening test. Features deriving from self-reported personal and family histories in the EHR indicating family history of cancer were also constructed using a binary encoder. These were combined with binary features indicating whether the patient had been a new patient in the past year, age group, race, and marital status, also derived from the EHR. A data dictionary with descriptions of the encoding used for subsequent machine learning experiments is located in the Supplementary Materials Table S2.

### Feature Selection

Feature selection was done in two stages. The first stage used the mutual information score for discrete and continuous data from the sci-kit learn package (11). The process was repeated 20 times to increase the stability of the features selected. Features with average mutual information about the target (mammogram completion) less than 0.001 were removed. This process was repeated 20 times and the mutual information was averaged to reduce variability during selection. The next stage of feature selection used reverse feature elimination where logistic regression to sequentially remove features one at a time with *P* values over 0.1, starting with features that had the highest *P* value.

### Modeling and Evaluation

A classification approach to modeling was used with support vector machines (SVM), decision trees (DT), random forests (RF), extra trees, logistic regressions (LR), principal component logistic regression (PC-LR), and negative matrix factorization logistic regression (NMF-LR). This set of models was selected to represent emblematic approaches typical in machine learning and covered decision tree methods, ensemble approaches (combining models) as well as linear models with and without data compression preprocessing. Model evaluation involved evaluating a series of metrics using 10-fold cross validation where the model was trained on 90% of the data with 10% held out for testing. Performance metrics were calculated and averaged across each test fold. These methods were implemented using the sci-kit learn package in python 3.8. Metrics used for evaluation included: area under the curve (AUC) of the receiver operating characteristic (ROC), harmonic mean of the precision and recall (F1), overall accuracy, positive predictive value (PPV), and negative predictive value (NPV). Significance level of 0.05 was used for all confidence intervals.

Top performing models were selected by AUC because it represented the balance between the true positive rate (where patients were not completing the mammogram with 180 days were correctly identified) and the false positive rate (where patients who completed screening, but incorrectly labeled by the model as likely not to complete). AUC was chosen as a primary metric because it describes the model's ability to discriminate between the cases and controls at various thresholds, and usage of the model can be tailored to the end users’ tolerance for false positives. The results of the 10-fold cross validation demonstrate how different types of models will perform on new (out of the box samples). A logistic regression model was trained to show coefficient effect sizes as well as p-values to quantify the contribution of individual features towards the model's prediction. An analysis of the patients in the 97.5 percentile used 20 random samples of 50% of the patients to demonstrate the model's ability to identify a small cohort of patients to which interventions can be focused and calculate performance metrics. A calibration table was also developed so that the model's PPV, NPV, and accuracy can be estimated on a range of cut points (thresholds) where predictions above the threshold are considered likely to fail to follow through with screening.

### Data Availability

This study was approved by the Medical University of South Carolina (MUSC) Internal Review Board (IRB Pro00101494). Patient data used for this study was de-identified for limited use and is not available for public distribution.

## Results

Study population demographics are shown in Table 1. Basic comparison of these characteristics indicates that African American race and marriage are associated with greater follow through with screening. Younger (55–60) and older age (70–74) on the contrary were associated with less follow through. From the MUSC EHR, during the study time frame, 3,490 female patients, aged 50–74 were identified as having a mammogram screening test ordered, of which 28.9% with confidence (27.4%–30.4%) failed to have a completed screening test in the following 180 days observed at MUSC. After removing patients where the test was ordered and completed on the same day, 1,826 patients were found to have met the selection criteria outlined in the methods with 961 cases (missing screening after ordered) and 865 controls (completing screening after ordered within 180 days) were identified and summarized in the Table 2. Summary statistics on the selected cohort showing the distribution of cases and controls with regard to demographic, insurance, comorbidity, and patient histories are shown in Table 3.

**TABLE 2:** Results of the study cohort selection

Cohort selection	Count	Proportion failed to complete screening
Female patients aged 50–74 with at least mammogram ordered in study time frame	3,940	28.9% with confidence (27.4%–30.4%)
Patients found to have a same day mammogram completion, history of breast cancer, or order was less than 180 days prior to the end of the study, or had a test with status “sent”, but completion could not be determined	2,114	
Cases (failed to complete screening)	961	
Controls (completed screening in within following 1–180 days)	856	
Total study cohort	1,826	52.6% with confidence (50.3%–54.9%)

**TABLE 3:** Summary statistics of MUSC cohort selected for study

Summary statistics	Variable name	Cases	Controls	Proportion among cases	Proportion among controls	OR	Fisher *P*
Totals	Patient count	961	865				
Billed or problem list diagnosis	F32 Depressive episode	32	11	(0.022–0.045)	(0.005–0.02)	2.632	0.005
	F41 Other anxiety disorders	30	10	(0.02–0.042)	(0.004–0.019)	2.714	0.006
	N95 Billed Menopause or perimenopause	11	1	(0.005–0.018)	(0.0–0.003)	9.952	0.007
	Z72 Problems related to lifestyle	13	3	(0.006–0.021)	(0.0–0.007)	3.921	0.024
	Anemia dx or pl	16	5	(0.009–0.025)	(0.001–0.011)	2.895	0.045
	Depression dx or pl	49	20	(0.037–0.065)	(0.013–0.033)	2.217	0.003
	Sleep dx or pl	36	15	(0.025–0.049)	(0.009–0.026)	2.171	0.011
Demographics	African-American	355	360	(0.339–0.4)	(0.383–0.449)	0.892	0.202
	Asian	7	7	(0.002–0.013)	(0.002–0.014)	0.905	1
	Divorced or Sep	168	130	(0.151–0.199)	(0.126–0.174)	1.169	0.235
	Married	452	448	(0.439–0.502)	(0.485–0.551)	0.913	0.273
	Single	239	186	(0.221–0.276)	(0.188–0.242)	1.163	0.178
	White-Caucasian	576	482	(0.568–0.63)	(0.524–0.59)	1.081	0.317
	Age under_60	417	292	(0.403–0.465)	(0.403–0.465)	1.292	0.004
	Age 60–65	215	181	(0.197–0.25)	(0.182–0.236)	1.075	0.543
	Age 65–70	222	217	(0.204–0.258)	(0.222–0.28)	0.926	0.49
	Age over_70	107	175	(0.091–0.131)	(0.176–0.229)	0.553	<0.001
Insurance	Managed care	31	16	(0.021–0.043)	(0.01–0.027)	1.753	0.076
	Medicaid	17	2	(0.009–0.026)	(0.0–0.006)	7.69	0.001
	Medicare	178	154	(0.161–0.21)	(0.153–0.204)	1.046	0.721
Patient History	Family History of breast cancer	111	122	(0.095–0.136)	(0.118–0.164)	0.823	0.165
	History of diabetes	75	68	(0.061–0.095)	(0.061–0.097)	0.998	1
	Neg family history of breast cancer	109	112	(0.093–0.133)	(0.107–0.152)	0.881	0.393
Previous Visits	Electrocardiogram prior 365	109	126	(0.093–0.133)	(0.122–0.169)	0.783	0.083
	Established patient prior 365	700	672	(0.7–0.757)	(0.749–0.805)	0.942	0.414
	Hemoglobin test prior 365	83	101	(0.069–0.104)	(0.095–0.138)	0.744	0.063
	New patient prior 365	260	181	(0.242–0.299)	(0.182–0.236)	1.3	0.015
	Pneumococcal vac prior 365	49	62	(0.037–0.065)	(0.054–0.089)	0.715	0.097
	Preventive visit prior 365	129	107	(0.113–0.156)	(0.102–0.146)	1.091	0.535
	Urinalysis prior 365	124	151	(0.108–0.15)	(0.149–0.2)	0.743	0.024

**FIGURE 1:**
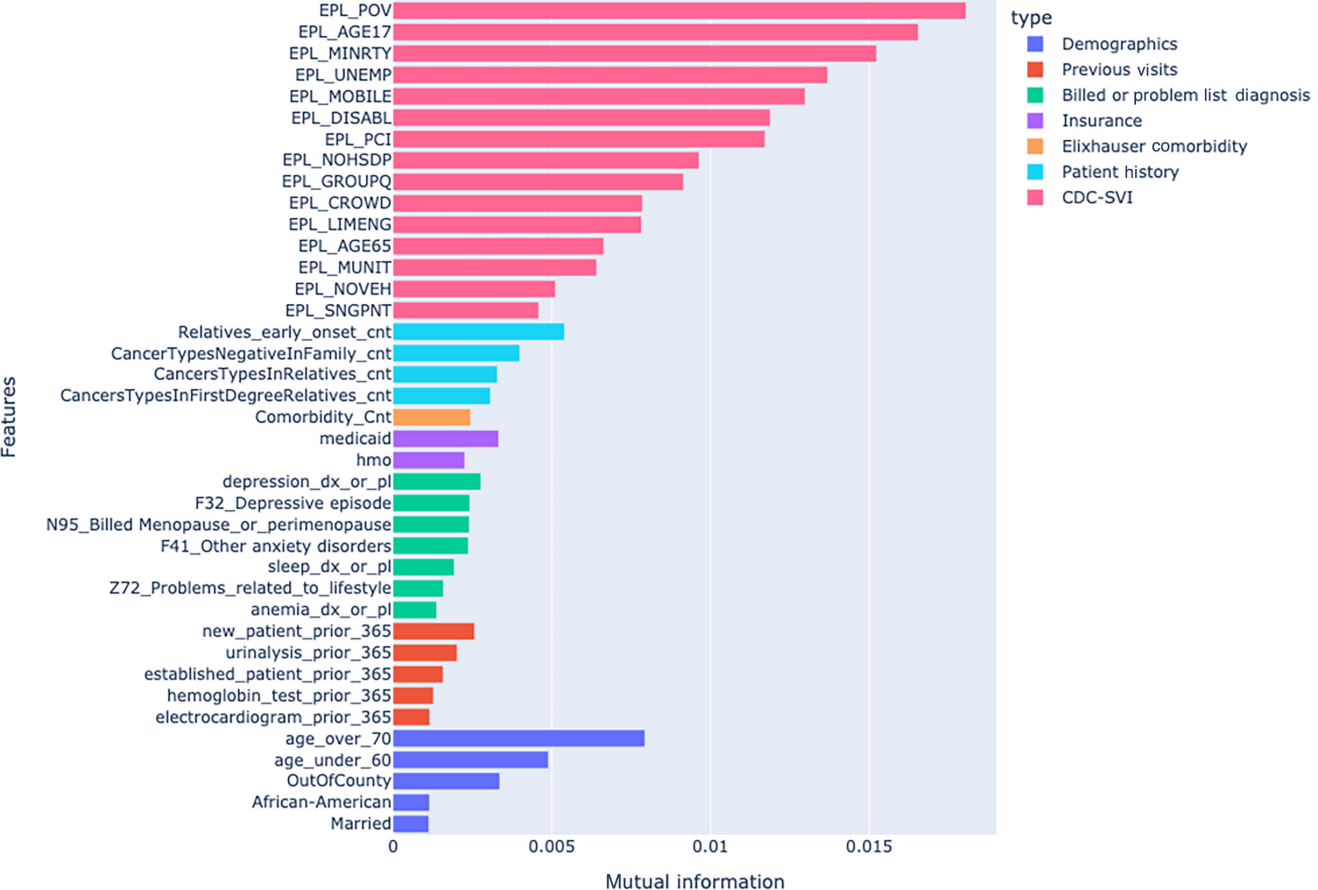
Mutual information of features with respect to failing to complete a breast cancer screening test in 180 days.

The original feature set, after feature construction, included 55 features summarizing comorbidity, insurance, demographics, diagnoses, patient and family history and prior visits. This was reduced to 39 features after selecting those with average mutual information about 0.001. The results of the selection can be seen in Figure 1, which shows the highest mutual information attributed to the percentile percentage of persons in poverty (EPL_POV) which estimates the relative percentile of the poverty rate by census track. The feature set was then further reduced to 13 after applying logistic regression and recursively removing the feature with the highest *P* value, until all *P* values were under 0.1. An added benefit of this process is that no highly correlated features remained in the curated features. Supplementary Figure SF1 shows a heat map of the correlations between selected features and is included in Supplementary Data.

**FIGURE 2:**
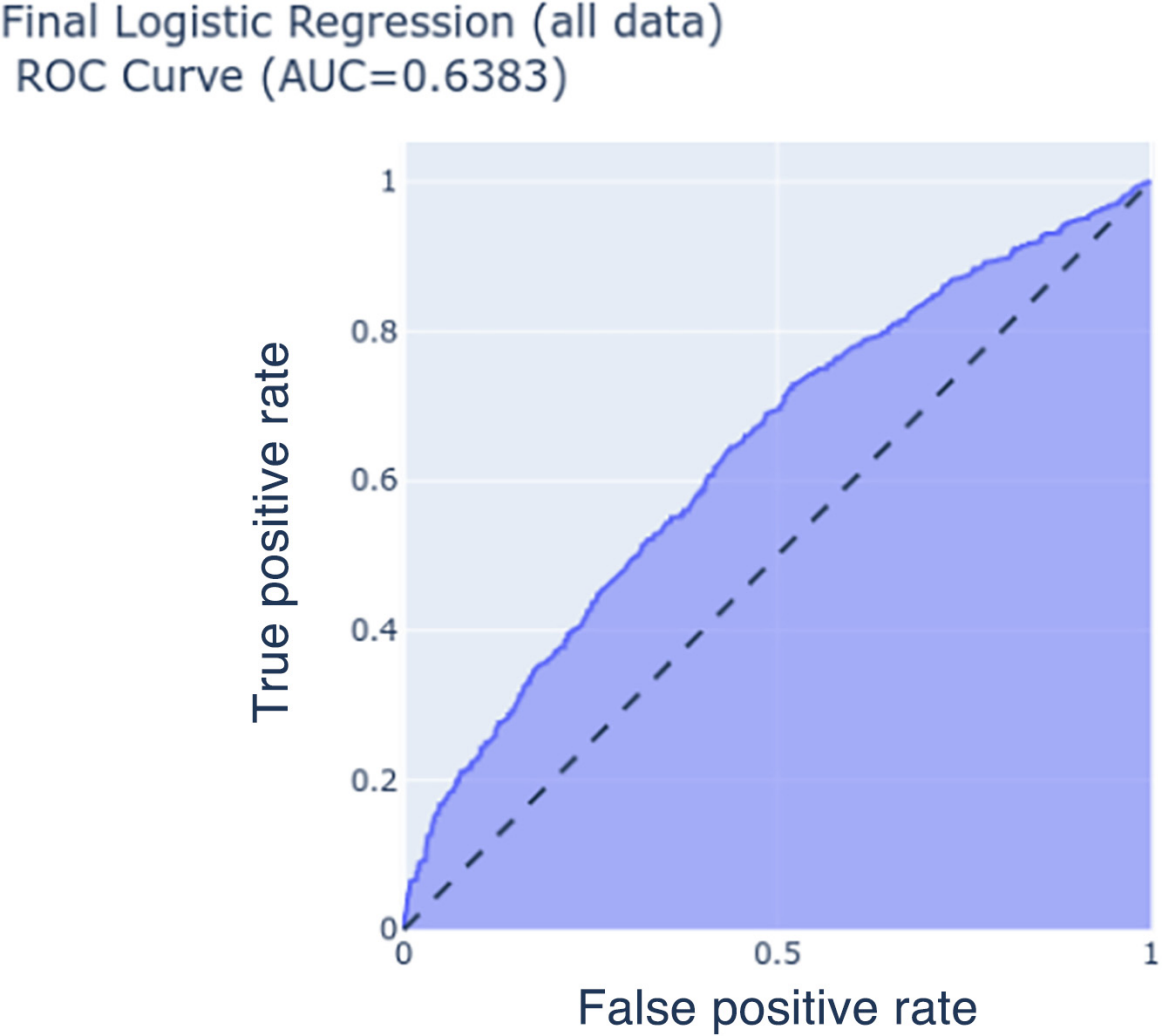
Receiver operating characteristic curve of logistic regression (build on all data) demonstrating the discriminating ability of the model.

Performance estimates of the different types of machine learning classifiers were estimated on the final set of selected features. Modeling types of logistic regression and logistic regression with PCA preprocessing had the highest average AUC across unseen test data of 0.63. The cross-validation results show limited or no improvement when using complex model types such as tree-based methods, ensembles, support vector machines or complex preprocessing such as PCA or NMF over traditional logistic regression. Performance results of the 10-fold cross validation experiment can be seen in Table 4. Logistic regression was chosen as the best classification method since other methods showed only slight improvements, and interpretation of the impact of each variable was limited. The final logistic regression model was trained only on the selected features, but on the entire dataset without regularization, and resulted in an AUC of 0.63 with confidence (0.589–0.683) and accuracy of 0.59 with confidence (0.569–0.619). The ROC for the final logistic regression is shown in Figure 2 and demonstrates how sensitivity and specificity change at different thresholds. Coefficients from the resulting regression are seen in Table 5, and a data dictionary for variables used in the final model is included in Supplementary Table S3.

**TABLE 4:** Cross-validated model training results on test fold showing logistic regression as the best performer by AUC, and SVM by F1 score

Classifier	AUC score test	F1 score test	Accuracy score test	NPV test	PPV test
Logistic Regression	0.626, SD (0.031)	0.638, SD (0.022)	0.594, SD (0.021)	0.584, SD (0.035)	0.601, SD (0.023)
Logistic Regression with PCA	0.626, SD (0.031)	0.636, SD (0.023)	0.594, SD (0.021)	0.584, SD (0.035)	0.603, SD (0.024)
Support Vector Machine-Linear	0.624, SD (0.031)	0.646, SD (0.023)	0.591, SD (0.026)	0.589, SD (0.039)	0.595, SD (0.03)
Logistic Regression with NMF	0.622, SD (0.03)	0.653, SD (0.026)	0.593, SD (0.025)	0.597, SD (0.045)	0.592, SD (0.024)
Support Vector Machine-rbf	0.6, SD (0.03)	0.609, SD (0.027)	0.579, SD (0.022)	0.56, SD (0.033)	0.596, SD (0.029)
Extra Trees	0.6, SD (0.024)	0.619, SD (0.02)	0.573, SD (0.022)	0.558, SD (0.03)	0.585, SD (0.028)
Random Forest	0.594, SD (0.026)	0.629, SD (0.022)	0.576, SD (0.023)	0.566, SD (0.032)	0.584, SD (0.027)
Decision Tree	0.554, SD (0.025)	0.592, SD (0.073)	0.538, SD (0.024)	0.53, SD (0.049)	0.552, SD (0.023)

**TABLE 5:** Coefficients on logistic regression predict failure to complete a breast cancer screening test, demonstrating effect of menopause-related diagnoses and Medicaid associated with increasing risk of missing a screening

Variable name	Coefficient	SE	z	P>|z|	Lower confidence	Upper confidence
N95 Billed Menopause or perimenopause	7.019	2.891	1.836	0.066	0.876	56.212
Medicaid	5.578	2.135	2.266	0.023	1.261	24.666
F41_Other anxiety disorders	1.931	1.470	1.708	0.088	0.908	4.111
EPL_DISABL	1.700	1.211	2.770	0.006	1.168	2.476
EPL_AGE17	1.514	1.201	2.266	0.023	1.058	2.168
EPL_MUNIT	1.428	1.151	2.537	0.011	1.084	1.880
Out Of County	1.390	1.161	2.202	0.028	1.037	1.864
age_under_60	1.231	1.109	2.006	0.045	1.005	1.507
Comorbidity Count	1.211	1.060	3.315	0.001	1.082	1.356
Married	0.749	1.098	−3.092	0.002	0.623	0.900
established_patient_prior_365	0.748	1.115	−2.679	0.007	0.604	0.925
African American	0.603	1.120	−4.470	0.000	0.483	0.753
age_over_70	0.546	1.154	−4.228	0.000	0.412	0.723

Of the binary features, having a prior billed diagnosis ICD10CM of N95 (Menopausal and other perimenopausal disorders) had the highest effect size with patients being 7 times with confidence (0.86–56.2) more likely to miss the screening test with *P* value 0.066. This is also supported by summary statistics where (1/864) controls and (11/950) cases had a N95 diagnoses with Fisher OR 10.0, *P* = 0.007. The next highest effect size was insurance of Medicaid, where patients are 5.5 times with confidence (1.21–24.6) more likely to miss screening if they have Medicaid insurance. Patients having a billed ICD10CM of F41 (other anxiety disorders) were 1.7 times with confidence (0.91–4.1) to miss screening.

Top contributors to indicating an increased likely hood that a patient follows through with screening are age over 70, race of African American, having a visit as an established patient in the last year, and having marital status of married. Given the prior feature selection step using mutual information and logistic regression, only the African American race category was selected as a feature, indicating the reference level for the coefficients is White or Caucasian as well as all other races. The association with African American race and being less likely to miss a screening test is also supported in the overall statistics, with controls having (360/505) African Americans versus all other races, and cases having (365/606) African Americans versus all other races with an OR of 8.22, *P* = 0.044.

Comorbidities also contributed, and the regression showed that an increase of one in the Elixhauser index was associated with 1.2 (1.05–1.51) increase in the risk of missing screening. The percentile of disabled persons (EPL_DISABL), percentile of persons under the age of 17 (EPL_AGE17) and percentile of persons in multi-unit housing (EPL_MUNIT) all were associated with increasing risk of missing screening.

**FIGURE 3:**
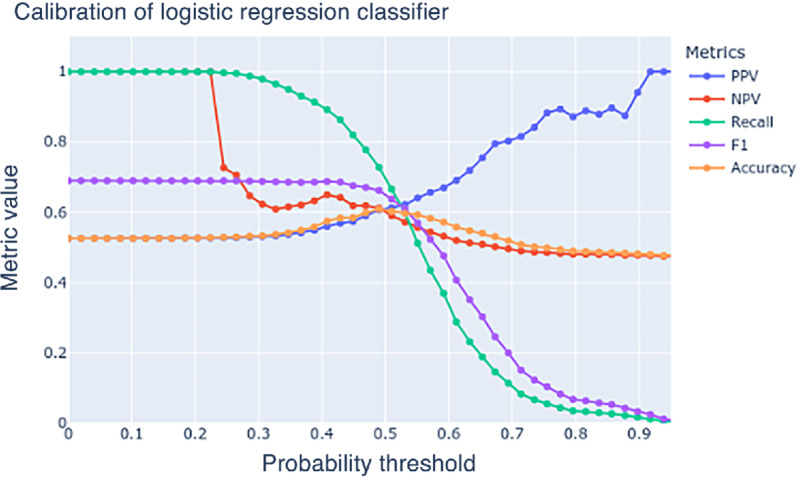
Calibration plot showing logistic regression metrics when probability threshold is changed.

Repeated sampling of the group of patients predicted by the model to be at the highest risk (97.5 percentile) for not completing screening, resulted in a mean PPV of 0.888 (SD, 0.003). This indicates that an intervention focused on the top 2.5% of patients most at risk for missing screening, would likely only have 11 of 100 patients who would have completed the screening without intervention. Calibration results that demonstrate the models PPV, NPV, and recall at range of thresholds shown in Figure 3. Tabulated results are available in the Supplementary Table S1.

## Discussion

Results from machine learning experiments on test folds during cross validation had a mean accuracy of 0.594 (0.022 SD) and AUC of 0.626 (0.031 SD) when logistic regression was applied. This demonstrates the model had discriminative ability to predict which patients will not follow through with screening. Although the results indicate the model is not likely to be powerful enough to be clinically useful for making predictions about individuals. It was demonstrated that a small group of patients could be identified with a very low likelihood if followed through. The importance of a patient's geographic location to breast cancer screening is clearly demonstrated, with the coefficient percentile percentage of disabled persons EPL_DISABLE variable. This effectively shows for every 10-percentile increase in the ranking on the community proportion of population living with a disability, the risk of missing a breast cancer screening test would also be expected to increase by 7% (1.6%–24.7%). Slightly lower effect sizes were observed in the EPL_AGE17 and EPL_MUNIT variables. Also, patients with addresses outside of the county had an increase in risk of missing a screening of 23.1% (0.5%–80.0%). It Is possible that this effect is a failure to capture screening tests conducted in health systems not affiliated with MUSC. The finding that African American patients are more likely to follow through with screening is also reflected in state-wide data that indicated 81.4% of women had a mammogram in the prior two years, in contrast to only 77.1% of White/ Caucasian patients in 2018 (5).

A primary limitation of this study is that the data was sourced from a single health system, MUSC. The confounding effect of screening tests ordered at MUSC but completed at another health system and not captured in MUSC's EHR is likely still present in the data. Masking “SENT” mammogram orders and patients with out of state addresses and insurance may have not totally mitigated the leakage. This may have contributed to coefficients fit by logistic regression associating patients with addresses out of state or out of county with decreased screening uptake. It is not clear for this study whether screening tests are occurring at other health systems and simply not being captured. This analysis carries the assumption that people have enough access to the health care system that they have office visits. It is unclear whether the incites of this study would apply to the general population, or only those with at least minimal access to a health system.

The purpose of this model development is to create an automated system to identify a small group of individuals with a high risk for nonadherence to screening. Because the model was trained on data that masked mammograms completed on the same day as ordered, and to be included in the data set, a mammogram screening would have had to been ordered by a health care provider, it follows that the model should be applied to patients following a visit where the screening test has been ordered. Focusing on the 2.5% of patients at highest risk for non-completion, could serve as an effective filter for choosing which patients might be eligible for high-cost expensive interventions such as arranging transportation, childcare, or discounting screening. Since the PPV is very high for this small group, care managers could have confidence that resources are being directed to groups of patients that are very unlikely to complete screening without them. The calibration table can be used by researchers or health systems to enter a specific metric that is most valued, and model thresholds and other metrics that would be expected are returned. For example, if a health system was implementing a low-cost intervention such as text message reminders, having a high recall may be required. Setting recall at 0.95, the resulting model threshold is 0.347 which would also result in PPV of 0.547. The effect of this would be that most patients receive a message, except for a small group of patients that were very likely to follow through with screening regardless. The recall indicates that 19 of 20 patients who would have otherwise missed screening will receive a message. A high-cost intervention such as arranging transportation may require high PPV. Setting PPV at 0.941 would result in recall 0.017. This indicates less than 2% of those who otherwise would have missed screening would receive the intervention. However, of those who did have transportation arranged, the recall indicates only roughly 1 in 20 would have completed the screening regardless of the intervention. Future work to improve the accuracy of mammogram screening follow through prediction should include increasing the sample size and diversity. This can be accomplished by training and testing models across multiple health systems. Supplementing existing data with additional sources such as clinical notes and claims may also lead to future improvements in accuracy.

### Conclusion

Predicting mammogram screening uptake remains a challenging problem. However, it can be estimated using EHR and geographic data using logistic regression. An increased risk of missing screening can be associated with geographic areas where patients live that have higher proportions of disabled persons, higher proportions of populations under that age of 17 and higher proportions of multi-unit housing. Menopause and anxiety disorders were also associated with increased risk of missing screening. Since this model relies on EHR and geographic data, it can be automated to stratify patients. By utilizing the model to focus on the patients at highest risk, care managers can identify a group of patients who are very unlikely to complete screening and target them with interventions to increase screening uptake.

## Supplementary Material

Supplementary Table 1 Logistic Regression Calibration showing model performance at various probability cutoff thresholds.

Supplementary Table 2 Data dictionary showing encoding of each feature prior to feature selection steps, along with a description of the variable.

Supplementary Table 3 Data dictionary after feature selection used in machine learning classification experiments as well as final logistic regression model.
