# Supplementary material for: Predicting Mammogram Screening Follow Through with Electronic Health Record and Geographically Linked Data
Source: Cancer Res Commun. 2023 Oct 19;3(10):2126–32. doi: 10.1158/2767-9764.CRC-23-0263 (PMC10586236; doi:10.1158/2767-9764.CRC-23-0263)
Supplement: Supplementary Table 1 — Logistic Regression Calibration showing model performance at various probability cutoff thresholds. [file crc-23-0263-s01.docx]

**Table ST1. Logistic Regression Calibration showing model performance at various probability cutoff thresholds.**

|  | **npv** | **ppv** | **accuracy** | **f1** | **recall** | **tp** | **fp** | **fn** | **tn** | **threshold** | **Predicted pos cnt** | **Predicted neg cnt** |
| --- | --- | --- | --- | --- | --- | --- | --- | --- | --- | --- | --- | --- |
| **8** | NaN | 0.526 | 0.526 | 0.690 | 1.000 | 961 | 865 | 0 | 0 | 0.163 | 1826 | 0 |
| **9** | 1.000 | 0.527 | 0.527 | 0.690 | 1.000 | 961 | 864 | 0 | 1 | 0.184 | 1825 | 1 |
| **10** | 1.000 | 0.527 | 0.527 | 0.690 | 1.000 | 961 | 864 | 0 | 1 | 0.204 | 1825 | 1 |
| **11** | 1.000 | 0.527 | 0.528 | 0.690 | 1.000 | 961 | 862 | 0 | 3 | 0.224 | 1823 | 3 |
| **12** | 0.727 | 0.528 | 0.529 | 0.690 | 0.997 | 958 | 857 | 3 | 8 | 0.245 | 1815 | 11 |
| **13** | 0.706 | 0.528 | 0.530 | 0.690 | 0.995 | 956 | 853 | 5 | 12 | 0.265 | 1809 | 17 |
| **14** | 0.647 | 0.530 | 0.532 | 0.689 | 0.988 | 949 | 843 | 12 | 22 | 0.286 | 1792 | 34 |
| **15** | 0.623 | 0.531 | 0.533 | 0.688 | 0.979 | 941 | 832 | 20 | 33 | 0.306 | 1773 | 53 |
| **16** | 0.609 | 0.533 | 0.537 | 0.687 | 0.965 | 927 | 812 | 34 | 53 | 0.327 | 1739 | 87 |
| **17** | 0.616 | 0.537 | 0.542 | 0.686 | 0.950 | 913 | 788 | 48 | 77 | 0.347 | 1701 | 125 |
| **18** | 0.621 | 0.542 | 0.549 | 0.685 | 0.931 | 895 | 757 | 66 | 108 | 0.367 | 1652 | 174 |
| **19** | 0.633 | 0.549 | 0.559 | 0.686 | 0.914 | 878 | 722 | 83 | 143 | 0.388 | 1600 | 226 |
| **20** | 0.650 | 0.560 | 0.575 | 0.688 | 0.892 | 857 | 672 | 104 | 193 | 0.408 | 1529 | 297 |
| **21** | 0.642 | 0.569 | 0.584 | 0.686 | 0.863 | 829 | 628 | 132 | 237 | 0.429 | 1457 | 369 |
| **22** | 0.619 | 0.574 | 0.585 | 0.676 | 0.820 | 788 | 584 | 173 | 281 | 0.449 | 1372 | 454 |
| **23** | 0.619 | 0.590 | 0.599 | 0.671 | 0.778 | 748 | 519 | 213 | 346 | 0.469 | 1267 | 559 |
| **24** | 0.612 | 0.607 | 0.609 | 0.662 | 0.728 | 700 | 453 | 261 | 412 | 0.490 | 1153 | 673 |
| **25** | 0.590 | 0.613 | 0.603 | 0.638 | 0.666 | 640 | 404 | 321 | 461 | 0.510 | 1044 | 782 |
| **26** | 0.572 | 0.622 | 0.597 | 0.610 | 0.599 | 576 | 350 | 385 | 515 | 0.531 | 926 | 900 |
| **27** | 0.557 | 0.641 | 0.593 | 0.569 | 0.512 | 492 | 275 | 469 | 590 | 0.551 | 767 | 1059 |
| **28** | 0.544 | 0.657 | 0.583 | 0.523 | 0.435 | 418 | 218 | 543 | 647 | 0.571 | 636 | 1190 |
| **29** | 0.532 | 0.670 | 0.572 | 0.476 | 0.369 | 355 | 175 | 606 | 690 | 0.592 | 530 | 1296 |
| **30** | 0.520 | 0.691 | 0.558 | 0.407 | 0.288 | 277 | 124 | 684 | 741 | 0.612 | 401 | 1425 |
| **31** | 0.513 | 0.719 | 0.548 | 0.351 | 0.232 | 223 | 87 | 738 | 778 | 0.633 | 310 | 1516 |
| **32** | 0.509 | 0.755 | 0.541 | 0.303 | 0.189 | 182 | 59 | 779 | 806 | 0.653 | 241 | 1585 |
| **33** | 0.502 | 0.795 | 0.531 | 0.246 | 0.146 | 140 | 36 | 821 | 829 | 0.673 | 176 | 1650 |
| **34** | 0.496 | 0.803 | 0.519 | 0.200 | 0.114 | 110 | 27 | 851 | 838 | 0.694 | 137 | 1689 |
| **35** | 0.490 | 0.816 | 0.508 | 0.151 | 0.083 | 80 | 18 | 881 | 847 | 0.714 | 98 | 1728 |
| **36** | 0.487 | 0.842 | 0.502 | 0.123 | 0.067 | 64 | 12 | 897 | 853 | 0.735 | 76 | 1750 |
| **37** | 0.486 | 0.883 | 0.499 | 0.104 | 0.055 | 53 | 7 | 908 | 858 | 0.755 | 60 | 1766 |
| **38** | 0.483 | 0.894 | 0.494 | 0.083 | 0.044 | 42 | 5 | 919 | 860 | 0.776 | 47 | 1779 |
| **39** | 0.481 | 0.872 | 0.490 | 0.068 | 0.035 | 34 | 5 | 927 | 860 | 0.796 | 39 | 1787 |
| **40** | 0.481 | 0.889 | 0.489 | 0.064 | 0.033 | 32 | 4 | 929 | 861 | 0.816 | 36 | 1790 |
| **41** | 0.480 | 0.879 | 0.487 | 0.058 | 0.030 | 29 | 4 | 932 | 861 | 0.837 | 33 | 1793 |
| **42** | 0.480 | 0.897 | 0.486 | 0.053 | 0.027 | 26 | 3 | 935 | 862 | 0.857 | 29 | 1797 |
| **43** | 0.478 | 0.875 | 0.484 | 0.043 | 0.022 | 21 | 3 | 940 | 862 | 0.878 | 24 | 1802 |
| **44** | 0.478 | 0.941 | 0.482 | 0.033 | 0.017 | 16 | 1 | 945 | 864 | 0.898 | 17 | 1809 |
| **45** | 0.477 | 1.000 | 0.480 | 0.025 | 0.012 | 12 | 0 | 949 | 865 | 0.918 | 12 | 1814 |
| **46** | 0.475 | 1.000 | 0.477 | 0.012 | 0.006 | 6 | 0 | 955 | 865 | 0.939 | 6 | 1820 |
| **47** | 0.475 | 1.000 | 0.476 | 0.008 | 0.004 | 4 | 0 | 957 | 865 | 0.959 | 4 | 1822 |
| **48** | 0.474 | 0.000 | 0.474 | 0.000 | 0.000 | 0 | 0 | 961 | 865 | 0.980 | 0 | 1826 |
