# Supplementary material for: Predicting Mammogram Screening Follow Through with Electronic Health Record and Geographically Linked Data
Source: Cancer Res Commun. 2023 Oct 19;3(10):2126–32. doi: 10.1158/2767-9764.CRC-23-0263 (PMC10586236; doi:10.1158/2767-9764.CRC-23-0263)
Supplement: Supplementary Table 2 — Data dictionary showing encoding of each feature prior to feature selection steps, along with a description of the variable. [file crc-23-0263-s02.docx]

**Table ST2. Data dictionary showing encoding of each feature prior to feature selection steps, along with a description of the variable.**

| **Variable Name** | **Variable Group** | **Description** | **Type** | **Min** | **Mean** | **Max** |
| --- | --- | --- | --- | --- | --- | --- |
| Comorbidity_Cnt | Elixhauser Comorbidity | Sum of Elixhauser Comorbidities | ordinal | 0 | 0.383 | 7 |
| White-Cauc | Demographics | 1 if patient race is White or Caucasian, otherwise 0 | binary | 0 | 0.579 | 1 |
| African-American | Demographics | 1 if patient race is African American, otherwise 0 | binary | 0 | 0.392 | 1 |
| Asian | Demographics | 1 if patient race is Asian, otherwise 0 | binary | 0 | 0.008 | 1 |
| Married | Demographics | 1 if patient martial status is married, otherwise 0 | binary | 0 | 0.493 | 1 |
| Single | Demographics | 1 if patient marital status is single,  otherwise 0 | binary | 0 | 0.233 | 1 |
| Divorced_or_Sep | Demographics | 1 if patient marital status is divorced or separated otherwise 0 | binary | 0 | 0.163 | 1 |
| OutOfCounty | Demographics | 1 if patient address was in a census tract outside of the county of the MUSC health system otherwise 0 | binary | 0 | 0.152 | 1 |
| EPL_POV | CDC-SVI | Census tract level percentile percentage of  persons below poverty estimate | continuous | 0.003 | 0.43 | 0.992 |
| EPL_UNEMP | CDC-SVI | Census tract level percentile percentage of  civilian (age 16+) unemployed estimate | continuous | 0 | 0.34 | 0.987 |
| EPL_PCI | CDC-SVI | Census tract level percentile per capita income  estimate | continuous | 0 | 0.359 | 0.963 |
| EPL_NOHSDP | CDC-SVI | Census tract level percentile percentage of  persons with no high school diploma (age 25+) estimate | continuous | 0 | 0.346 | 0.978 |
| EPL_AGE65 | CDC-SVI | Census tract level percentile percentage of persons aged 65 and older estimate | continuous | 0.007 | 0.541 | 0.996 |
| EPL_AGE17 | CDC-SVI | Census tract percentile percentage of persons aged 17 and younger estimate | continuous | 0.004 | 0.329 | 0.96 |
| EPL_DISABL | CDC-SVI | Census tract percentile percentage of civilian noninstitutionalized population with a disability estimate | continuous | 0.009 | 0.393 | 0.993 |
| EPL_SNGPNT | CDC-SVI | Census tract Percentile percentage of single parent households with children under 18 estimate | continuous | 0 | 0.369 | 0.997 |
| EPL_MINRTY | CDC-SVI | Census tract percentile percentage minority (all persons except white, non-Hispanic) estimate | continuous | 0 | 0.486 | 0.94 |
| EPL_LIMENG | CDC-SVI | Census tract percentile percentage of persons (age 5+) who speak English "less than well" estimate | continuous | 0 | 0.282 | 0.946 |
| EPL_MUNIT | CDC-SVI | Census tract percentile percentage housing in structures with 10 or more units estimate | continuous | 0 | 0.533 | 0.954 |
| EPL_MOBILE | CDC-SVI | Census tract percentile percentage mobile homes estimate | continuous | 0 | 0.562 | 0.998 |
| EPL_CROWD | CDC-SVI | Census tract percentile percentage households with more people than rooms estimate | continuous | 0 | 0.306 | 0.936 |
| EPL_NOVEH | CDC-SVI | Census tract percentile percentage households with no vehicle available estimate | continuous | 0 | 0.399 | 0.963 |
| EPL_GROUPQ | CDC-SVI | Census tract percentile percentage of persons in group quarters estimate1 | continuous | 0 | 0.365 | 0.992 |
| managed_care | Insurance | managed care patient insurance plan 1, otherwise 0 | binary | 0 | 0.026 | 1 |
| medicare | Insurance | Medicare patient insurance 1, otherwise 0 | binary | 0 | 0.182 | 1 |
| medicaid | Insurance | Medicaid patient insurance 1, otherwise 0 | binary | 0 | 0.01 | 1 |
| ppo | Insurance | Patient PPO insurance 1, otherwise 0 | Insurance | 0 | 0 | 0 |
| hmo | Insurance | Patient had HMO Insurance 1, otherwise 0 | Insurance | 0 | 0.014 | 1 |
| established_patient_prior_365 | Previous Visits | patient had a visit as an established patient in the prior 365 days 1, otherwise 0 | binary | 0 | 0.751 | 1 |
| new_patient_prior_365 | Previous Visits | patient had a visit as a new patient in the prior 365 days 1, otherwise 0 | binary | 0 | 0.242 | 1 |
| preventive_visit_prior_365 | Previous Visits | patient had a preventative care visit in the prior 365 days 1, otherwise 0 | binary | 0 | 0.129 | 1 |
| no_charge_cpt | Previous Visits | patient previously had a CPT Code with no charge 1, otherwise 0 | binary | 0 | 0.035 | 1 |
| office_visit_prior_365 | Previous Visits | patient had any office visit in the prior 365 days 1, otherwise 0 | binary | 0 | 0.912 | 1 |
| urinalysis_prior_365 | Previous Visits | patient had a urinalysis CPT code in the prior 365 then 1, otherwise 0 | binary | 0 | 0.151 | 1 |
| hemoglobin_test_prior_365 | Previous Visits | patient had a hemoglobin test CPT code in the prior 365 then 1, otherwise 0 | binary | 0 | 0.101 | 1 |
| electrocardiogram_prior_365 | Previous Visits | patient had a EKG CPT code in the prior 365 then 1, otherwise 0 | binary | 0 | 0.129 | 1 |
| pneumococcal vac_prior_365 | Previous Visits | patient had a pneumococcal vaccine in the prior 365 then 1, otherwise 0 | binary | 0 | 0.061 | 1 |
| age_under_60 | Demographics | 1 if 50<= patient age <60 otherwise 0 | binary | 0 | 0.388 | 1 |
| age_60-65 | Demographics | 1 if 60<= patient age <65 otherwise 0 | binary | 0 | 0.217 | 1 |
| age_65-70 | Demographics | 1 if 65 <= patient age <70 otherwise 0 | binary | 0 | 0.24 | 1 |
| age_over_70 | Demographics | 1 if 70<= patient age <75 otherwise 0 | binary | 0 | 0.154 | 1 |
| depression_dx_or_pl | Billed or Problem List Diagnosis | 1 if had patient had elixhausrer comorbidity of depression, or problem list indicated depression, otherwise 0 | binary | 0 | 0.038 | 1 |
| F41_Other anxiety disorders | Billed or Problem List Diagnosis | 1 if patient had a billed ICD10cm of F41, or anxiety indicated in the problem list, otherwise 0 | binary | 0 | 0.022 | 1 |
| N95_Billed Menopause_or_perimenopause | Billed or Problem List Diagnosis | 1 if patient had a billed ICD10cm of N95, otherwise 0 | binary | 0 | 0.007 | 1 |
| F32_Depressive episode | Billed or Problem List Diagnosis | 1 if patient had a billed ICD10cm of F32, otherwise 0 | binary | 0 | 0.024 | 1 |
| Z72_Problems_related_to_lifestyle | Billed or Problem List Diagnosis | 1 if patient had a billed ICD10cm of Z72, otherwise 0 | binary | 0 | 0.009 | 1 |
| anemia_dx_or_pl | Billed or Problem List Diagnosis | 1 if patient had an elixhauser combability indicating anemia, or anemia was indicated on the problem list, otherwise 0 | binary | 0 | 0.012 | 1 |
| sleep_dx_or_pl | Billed or Problem List Diagnosis | 1 if patient had a billed ICD10cm of G47 or sleep disorder indicated on the problem list, otherwise 0 | binary | 0 | 0.028 | 1 |
| CancerTypesNegativeInFamily_cnt | Patient History | Count of cancer types reported by the patient that have no family history | ordinal | 0 | 0.229 | 5 |
| CancersTypesInFirstDegreeRelatives_cnt | Patient History | Count of cancer types reported by the patient in parents, siblings, and children | ordinal | 0 | 0.583 | 6 |
| CancersTypesInRelatives_cnt | Patient History | Count of cancer types reported by the patient in all relatives | ordinal | 0 | 0.864 | 6 |
| Relatives_early_onset_cnt | Patient History | Count of relatives with early onset cancer reported by the patient | ordinal | 0 | 0.033 | 2 |
| FH_breast_cancer | Patient History | Patient reported family history of breast cancer 1, otherwise 0 | binary | 0 | 0.128 | 1 |
| hist_of_diabetes | Patient History | 1 if any Elixhasuser diabetes comorbidity, or indications on the problem list, otherwise 0 | binary | 0 | 0.078 | 1 |
| neg_FH_breast_cancer | Patient History | Patient reported having a negative family history of breast cancer 1, otherwise 0 | binary | 0 | 0.121 | 1 |
