# Supplementary material for: Predicting Mammogram Screening Follow Through with Electronic Health Record and Geographically Linked Data
Source: Cancer Res Commun. 2023 Oct 19;3(10):2126–32. doi: 10.1158/2767-9764.CRC-23-0263 (PMC10586236; doi:10.1158/2767-9764.CRC-23-0263)
Supplement: Supplementary Table 3 — Data dictionary after feature selection used in machine learning classification experiments as well as final logistic regression model. [file crc-23-0263-s03.docx]

**Table ST3. Data dictionary after feature selection used in machine learning classification experiments as well as final logistic regression model.**

| **Variable Name** | **Variable Group** | **Description** | **Type** | **Min** | **Mean** | **Max** |
| --- | --- | --- | --- | --- | --- | --- |
| Comorbidity_Cnt | Elixhauser Comorbidity | Sum of Elixhauser Comorbidities | ordinal | 0 | 0.383 | 7 |
| African-American | Demographics | 1 if patient race is African American, otherwise 0 | binary | 0 | 0.392 | 1 |
| Married | Demographics | 1 if patient martial status is married, otherwise 0 | binary | 0 | 0.493 | 1 |
| OutOfCounty | Demographics | 1 if patient address was in a census tract outside of the county of the MUSC health system otherwise 0 | binary | 0 | 0.152 | 1 |
| EPL_AGE17 | CDC-SVI | Census tract percentile percentage of persons aged 17 and younger estimate | continuous | 0.004 | 0.329 | 0.96 |
| EPL_DISABL | CDC-SVI | Census tract percentile percentage of civilian noninstitutionalized population with a disability estimate | continuous | 0.009 | 0.393 | 0.993 |
| EPL_MUNIT | CDC-SVI | Census tract percentile percentage housing in structures with 10 or more units estimate | continuous | 0 | 0.533 | 0.954 |
| medicaid | Insurance | Medicaid patient insurance 1, otherwise 0 | binary | 0 | 0.01 | 1 |
| established_patient_prior_365 | Previous Visits | patient had a visit as an established patient in the prior 365 days 1, otherwise 0 | binary | 0 | 0.751 | 1 |
| age_over_70 | Demographics | 1 if 70<= patient age <75 otherwise 0 | binary | 0 | 0.154 | 1 |
| F41_Other anxiety disorders | Billed or Problem List Diagnosis | 1 if patient had a billed ICD10cm of F41, or anxiety indicated in the problem list, otherwise 0 | binary | 0 | 0.022 | 1 |
| N95_Billed Menopause_or_perimenopause | Billed or Problem List Diagnosis | 1 if patient had a billed ICD10cm of N95, otherwise 0 | binary | 0 | 0.007 | 1 |
